# Five-gene signature predicts acute kidney injury in early kidney transplant patients

**DOI:** 10.18632/aging.203962

**Published:** 2022-03-23

**Authors:** Xia Zhai, Hongqiang Lou, Jing Hu

**Affiliations:** 1 Medical Molecular Biology Laboratory, School of Medicine, Jinhua Polytechnic, Jinhua 321000, China

**Keywords:** acute kidney injury, 5-gene signature, AUC, weighted co-expression network, support vector machine

## Abstract

Patients with acute kidney injury (AKI) show high morbidity and mortality, and a lack of effective biomarkers increases difficulty in its early detection. Weighted gene co-expression network analysis (WGCNA) detected a total of 22 gene modules and 6 miRNA modules, of which 4 gene modules and 3 miRNA modules were phenotypically co-related. Functional analysis revealed that these modules were related to different molecular pathways, which mainly involved PI3K-Akt signaling pathway and ECM-receptor interaction. The brown modules related to transplantation mainly involved immune-related pathways. Finally, five genes with the highest AUC were used to establish a diagnosis and prediction model of AKI. The model showed a high area under curve (AUC) in the training set and validation set, and their prediction accuracy for AKI was as high as 100%. Similarly, the prediction accuracy of AKI after 24 h in the 0 h transplant sample was 100%. This study may provide new features for the diagnosis and prediction of AKI after kidney transplantation, and facilitate the diagnosis and drug development of AKI in kidney transplant patients.

## INTRODUCTION

Acute kidney injury (AKI) is a common clinical syndrome with a high morbidity and mortality rate [1, 2]. The incidence of AKI is steadily increasing in recent years. Its incidence varies in different clinical settings such as cardiac surgery, intensive care units, and community settings [3]. For hospitalized patients, depending on the diagnostic criteria, the incidence of AKI can reach 13% [4]. The prevalence of AKI in patients undergoing cardiac surgery is about 35% [5]. Though prevention strategies, patient classification and technology have been greatly improved, AKI still has a high morbidity and mortality rate, especially for those in the intensive care unit (ICU), where the morbidity can reach 50–70%. Patients survived from AKI demonstrate a significant risk of developing chronic diseases (chronic kidney disease (CKD)) or a rapid development into end-stage renal diseases [6]. Early diagnosis is therefore important for early intervention, which could improve the prognosis of patients with AKI.

Serum creatinine (SCr), which is a marker of renal function, shows importance in assessing glomerular filtration rate (GFR) and is insensitive to acute changes in renal functions [7, 8]. There have been many studies aiming to find early biomarkers for AKI diagnosis. Parikh CR et al. determined kidney injury molecule-1 and liver fatty acid-binding protein as biomarkers of AKI [9]. Meersch M et al. found that TIMP-2, IGFBP7 can predict AKI early after cardiac surgery [10]. Using urinary exocrine, ZHANG et al. identified miRNA-30c-5p and miRNA-192-5p as potential biomarkers to renal injury caused by ischemia-reperfusion [11]. However, so far, reliable biomarkers sensitive to AKI and specific to its etiology are limited.

The aim of this study was to integrate miRNA and mRNA expression profile data from kidney transplantation to study altered miRNA and gene expression patterns between kidney transplant recipients and stable transplant recipients and to further identify AKI-related biomarkers. This study determined specific genes as potential biomarkers based on weighted co-expression networks and to further establish an AKI diagnostic and predictive classifier.

## RESULTS

### Identification of AKI-related co-expression modules by weighted co-expression analysis

**Figure 1:**
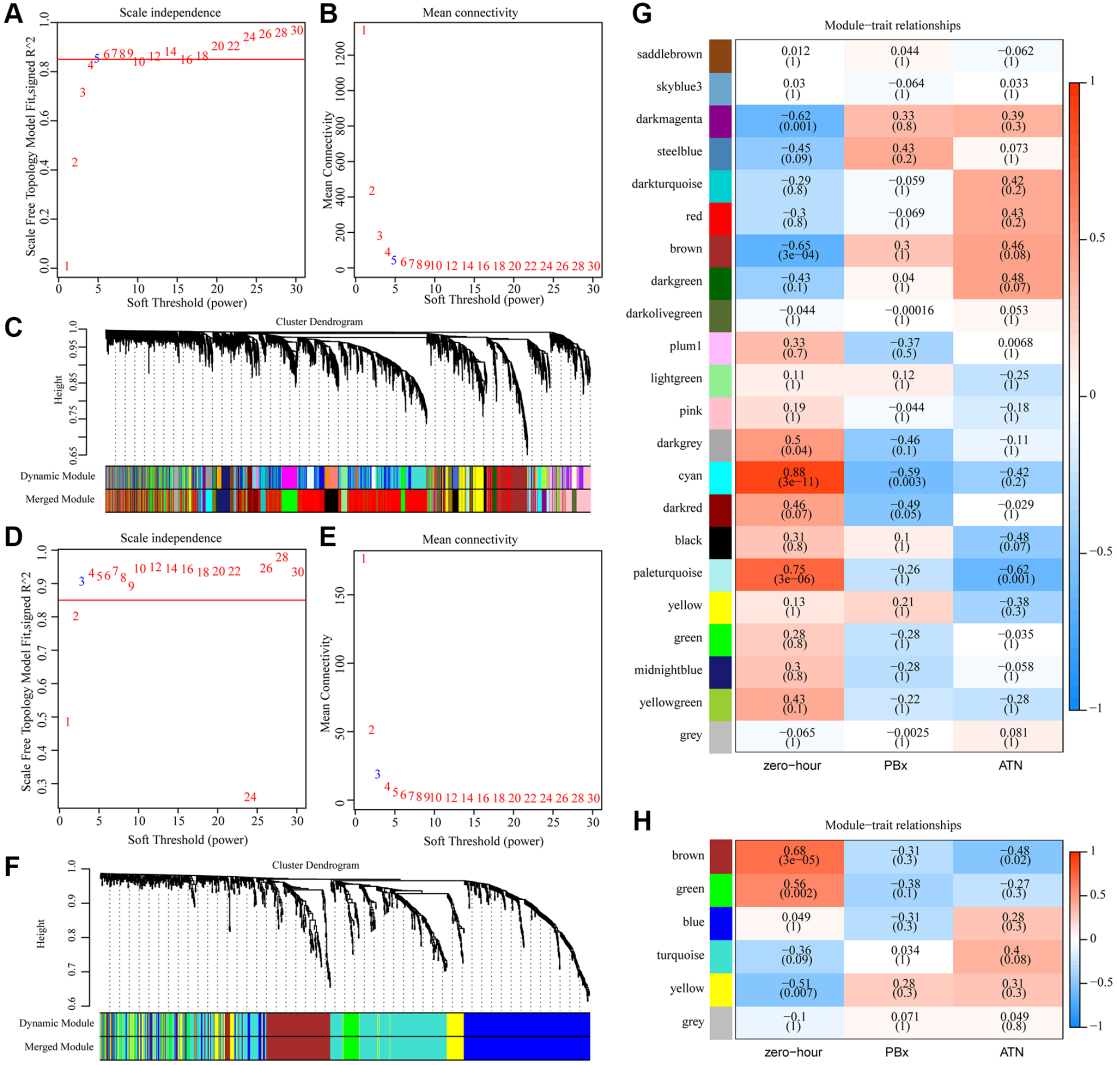
**Identification of AKI-related co-expression modules.** (**A**) Analysis of the scale-free fit index for various soft-thresholding powers (β) in mRNA expression profiles. (**B**) Analysis of the mean connectivity for various soft-thresholding powers in mRNA expression profiles. (**C**) Dendrogram of genes clustered based on a dissimilarity measure (1-TOM). (**D**) Analysis of the scale-free fit index for various soft-thresholding powers (β) in miRNA expression profiles. (**E**) Analysis of the mean connectivity for various soft-thresholding powers in mRNA expression profiles. (**F**) Dendrogram of miRNA clustered based on a dissimilarity measure (1-TOM). (**G**) Heat map of correlation between gene co-expression module and AKI. (**H**) Heat map of correlation between miRNA co-expression module and AKI.

To better screen AKI-related genes and miRNAs, Median Absolute Deviation (MAD) >25% was selected using gene expression profiles and miRNA expression profiles. Weighted co-expression networks were constructed using WGCNA, and for mRNA expression profiles, power of β = 5 (scale-free R^2 = 0.85) was the soft threshold to ensure a scale-free network (Figure 1A–1B). A total of 22 modules were identified (Figure 1C). Similarly, for miRNA expression profiles, the power of β = 3 (scale-free R^2 = 0.9) was a soft threshold to ensure a scale-free network (Figure 1D–1E). A total of 6 modules were identified (Figure 1F). Furthermore, the correlation between each gene module and AKI was analyzed according to the feature vectors of the modules. It was found that pale turquoise module was significantly negatively correlated with AKI but positively correlated with zero-hour, and that cyan module was significantly negatively correlated with PBx but positively correlated with zero-hour, moreover, brown and dark magenta module were significantly negatively correlated with zero-hour in the co-expression modules (Figure 1G). Among the six miRNA co-expression modules, three modules were closely correlated with zero-hour, among which the brown module was significantly negatively correlated with AKI (Figure 1H). These results indicated that abnormal transcriptome changes after kidney transplantation may lead to different clinical outcomes of kidney transplant patients.

### Functional analysis of phenotypic related gene co-expression modules

**Figure 2:**
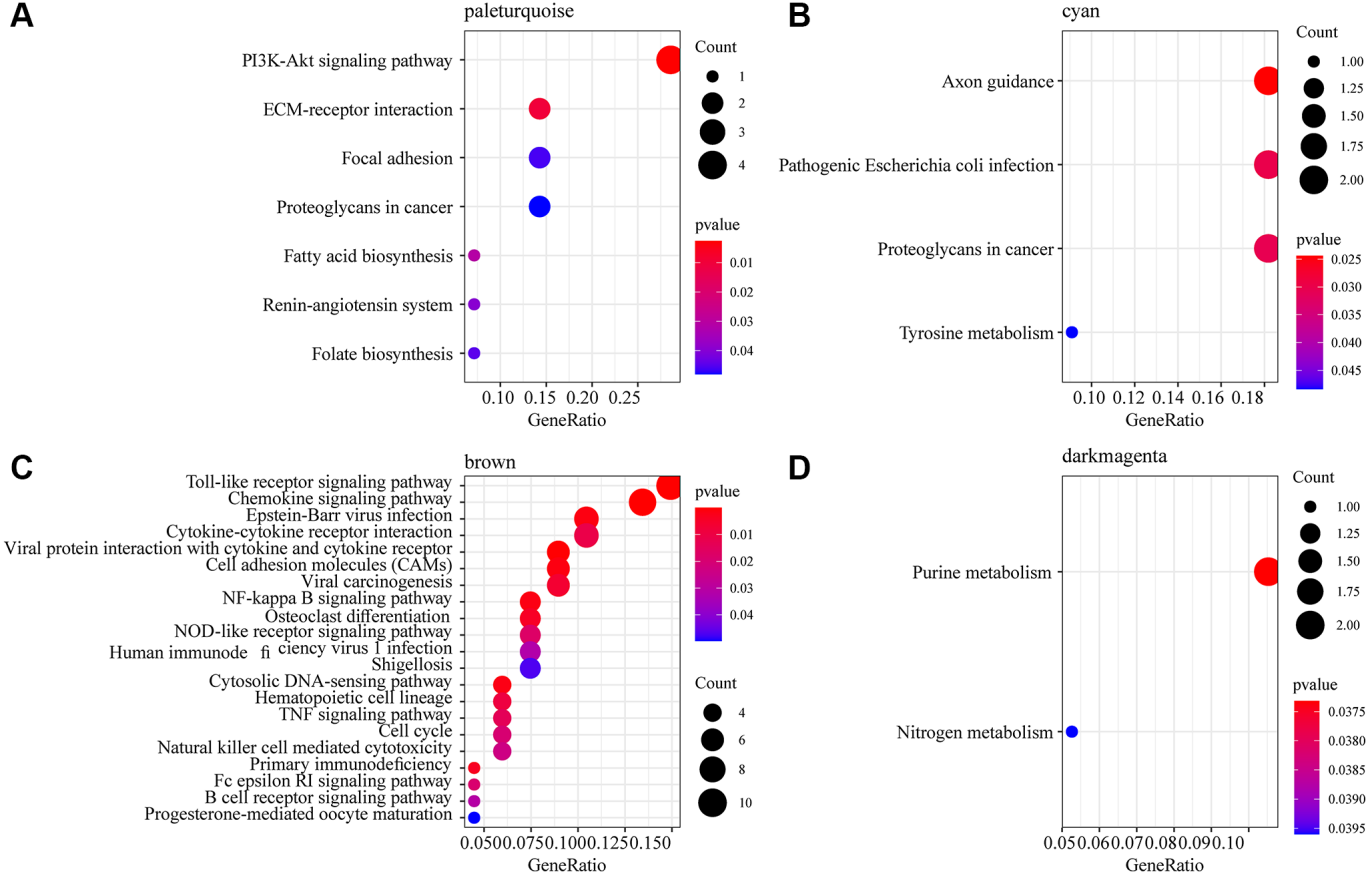
**Functional enrichment analysis of phenotype-related gene co-expression modules.** (**A**) 7 KEGG Pathway enriched by pale turquoise module. (**B**) 4 KEGG Pathway enriched by cyan module. (**C**) 21 KEGG Pathway enriched by brown module. (**D**) 2 KEGG Pathway enriched by dark magenta module. The dots are the number of genes enriched in the pathway; the color represents the significance of enrichment, the horizontal axis represents the enrichment factor, and the vertical axis represents the KEGG Pathway.

To observe the function of the three phenotype-related modules zero-hour, PBx and AKI, we extracted the most module-related genes (correlation coefficient > 0.8) from the four modules brown, dark magenta, pale turquoise and cyan, respectively. This study detected 136 genes in the brown module, 34 genes in the dark magenta module, 30 genes in the cyan module, and 30 genes in the pale turquoise module. After subjecting these genes to KEGG Pathway enrichment analysis, specifically, the pale turquoise module was enriched to seven KEGG Pathways, which were mainly related to the PI3K-Akt signaling pathway, ECM-receptor interaction, Fatty acid biosynthesis, and Renin-angiotensin system (Figure 2A); the cyan module was enriched to four Pathways, which were mainly related to Axon guidance, Pathogenic *Escherichia coli* infection (Figure 2B); the brown module was mainly enriched in immune-related pathways such as Toll-like receptor signaling pathway, Chemokine signaling pathway, Cytokine-cytokine receptor interaction (Figure 2C); the dark magenta module was enriched to the Purine metabolism and Nitrogen metabolism pathways (Figure 2D). Among the pathways enriched by these four modules, only Proteoglycans in cancer was enriched by dark magenta and cyan at the same time, and other pathways did show overlaps, indicating that different co-expression modules may participate in different biological pathways. For example, the brown module was enriched to a variety of immune-related pathways, and the genes in the brown module were significantly negatively correlated with kidney transplantation. This showed that the expression of genes in the brown module was mainly related to mid- and long-term immunosuppressive therapy in kidney transplant patients.

### MiRNA and mRNA specifically expressed in AKI

**Figure 3:**
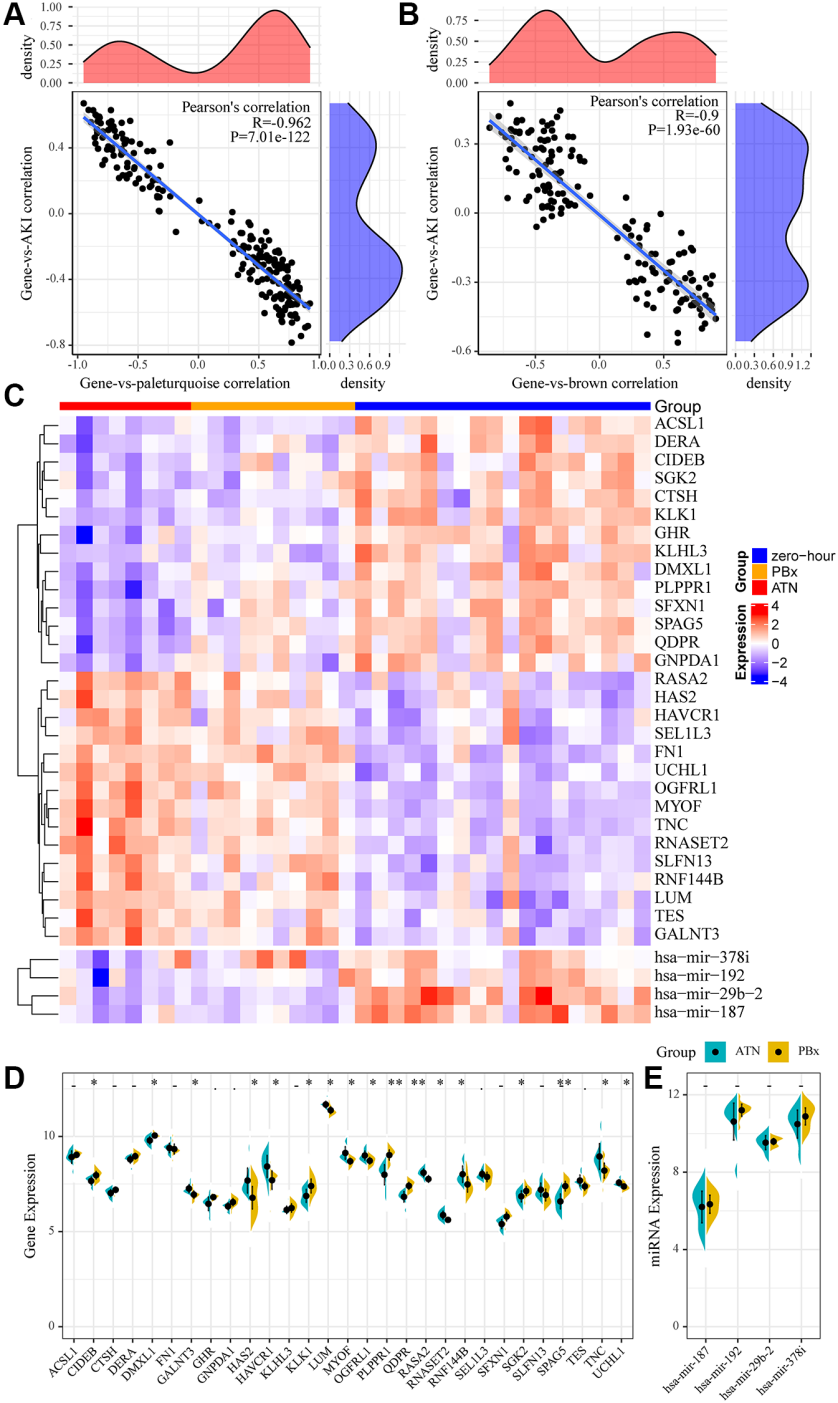
**miRNA and mRNA specifically expressed in AKI.** (**A**) Relationship between gene expression in pale turquoise module and module/AKI. The horizontal axis represents the correlation coefficient between gene expression and pale turquoise module, and the vertical axis represents the correlation coefficient between gene expression and AKI. (**B**) Relationship between miRNA expression in brown module and module/AKI. The horizontal axis represents the correlation coefficient between miRNA expression and brown module, and the vertical axis represents the correlation coefficient between miRNA expression and AKI. (**C**) heatmap of genes and miRNAs specifically expressed in the module. (**D**, **E**): Differences between genes and miRNAs specifically expressed in the module in AKI samples and PBx samples. Statistical *p*-values were obtained using the *t*-test, with “*” indicating *p* < 0.05 and “**” indicating *p* < 0.01, “.” indicates *p* < 0.1, “-” indicates *p* > 0.1.

We selected the most relevant gene modules and miRNA modules of AKI for further analysis to identify miRNAs and mRNAs specifically expressed in AKI. Here, 215 genes were included in the most relevant gene module of AKI (pale turquoise), which showed a double-peak distribution of correlations with AKI (Figure 3A). A total of 164 miRNAs were included in the most relevant miRNA module of AKI (brown), and the correlation of these miRNAs with AKI also showed a double-peak distribution (Figure 3B), indicating that the genes and miRNAs in the module presented two expression patterns in the AKI samples. We selected genes and miRNAs with expression correlation coefficients greater than 0.8 with the modules and greater than 0.4 with AKI as the genes and miRNAs specifically expressed in AKI. Here, we obtained 4 miRNAs and 29 genes. Fourteen of these 29 genes were significantly under-expressed in AKI and PBx samples, 15 genes were significantly overexpressed in AKI and PBx samples, and four miRNAs were significantly under-expressed in both AKI and PBx samples (Figure 3C). Furthermore, after comparing the expression differences of these genes in AKI and PBx samples, we found that 17(58.6%) of the genes showed significant expression differences (Figure 3D), indicating that these genes may serve as specific biomarkers for AKI. Interestingly, no significant difference in the expression of the four miRNAs was detected in the samples of AKI and PBx (Figure 3E). However, KEGG Pathway enrichment analysis of four miRNA target genes showed a total of 87 enriched pathways, which had the most intersections with the KEGG Pathway enriched by the brown and pale turquoise modules of co-expressed genes (Supplementary Figure 1A), suggesting that these four miRNAs may be involved in both AKI and immunomodulatory processes.

### Identification of key mRNA markers in AKI

**Figure 4:**
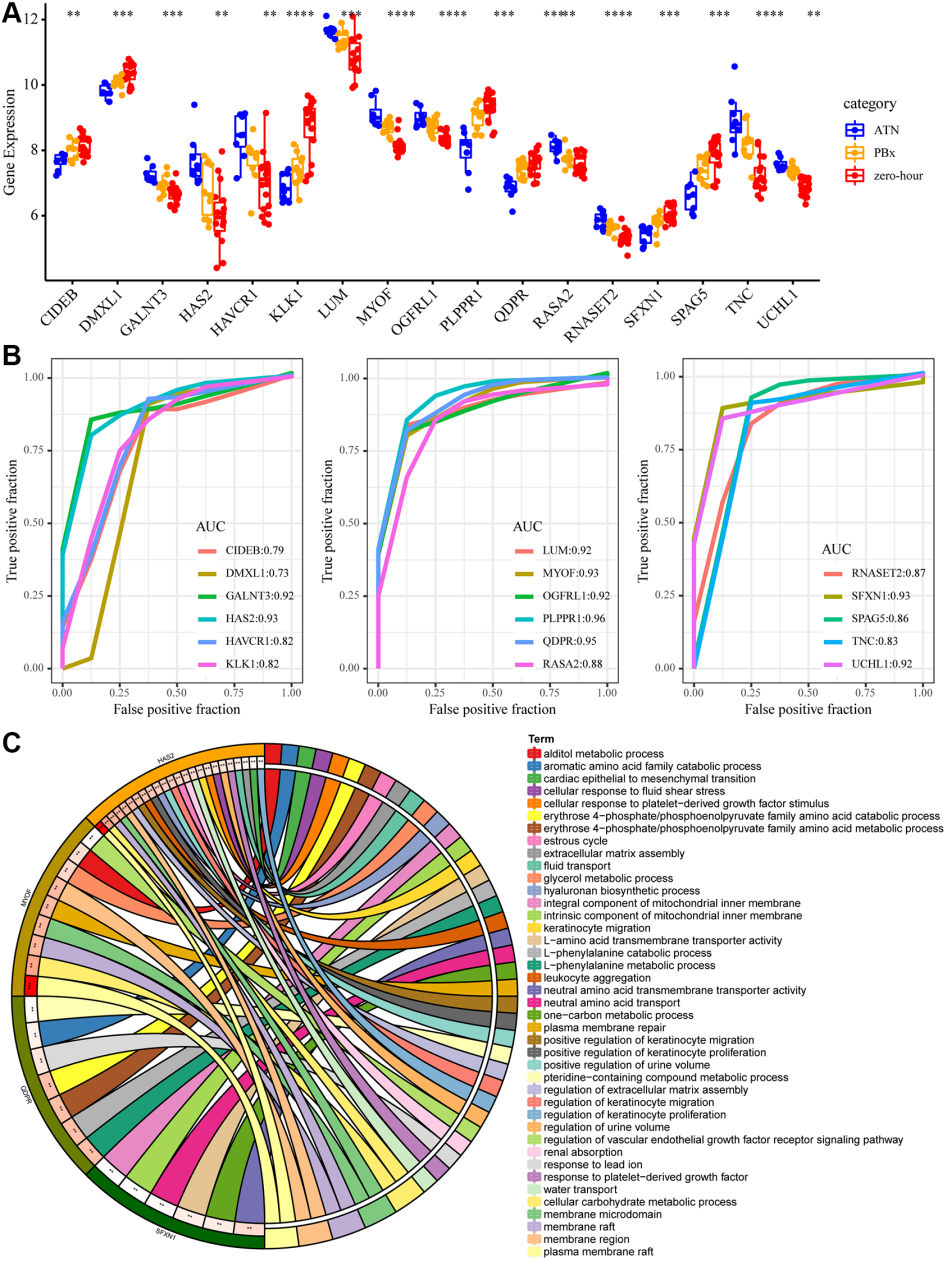
**Key mRNA biomarkers in AKI.** (**A**) The expression differences of the 17 genes in AKI, PBX and zero-hour, among which “*” means *p* < 0.05; “**” said *p* < 0.01; “***” means *p* < 0.001, “****” means *p* < 0.0001. (**B**) ROC curve of 17 genes. (**C**) The enrichment results of GO function of the five largest AUC genes. Different colors on the right side of the circle represent the GO Term, different colors on the left side of the circle represent genes, and the lines represent gene enrichment to the corresponding GO Term.

To further screen gene markers in AKI, we selected 17 genes (10 specifically high-expressed and 7 genes specifically low-expressed) differences in the three types of samples (Figure 4A). Support vector machines were used to establish an AKI prediction model based on the expression of each gene, and ROC was used to analyze the prediction performance of each gene (Figure 4B). The results showed that AUC of 9(53%) genes was greater than 0.9 and AUC of 15 (88%) genes was greater than 0.8, showing a high performance in predicting AKI samples based on the expression of these genes. Subsequently, the top five AUC genes (HAS2, MYOF, PLPPR1, QDPR, SFXN1) were subjected to GO enrichment analysis, with FDR <0.01 as the threshold. Four genes were found to be enriched to 41 GO terms (Figure 4C), which were mainly related to transmembrane transport, amino acid metabolism, renal absorption and positive regulation of urine volume, suggesting that these four genes may play an important role in kidney injury.

### Construction of diagnostic model

**Figure 5:**
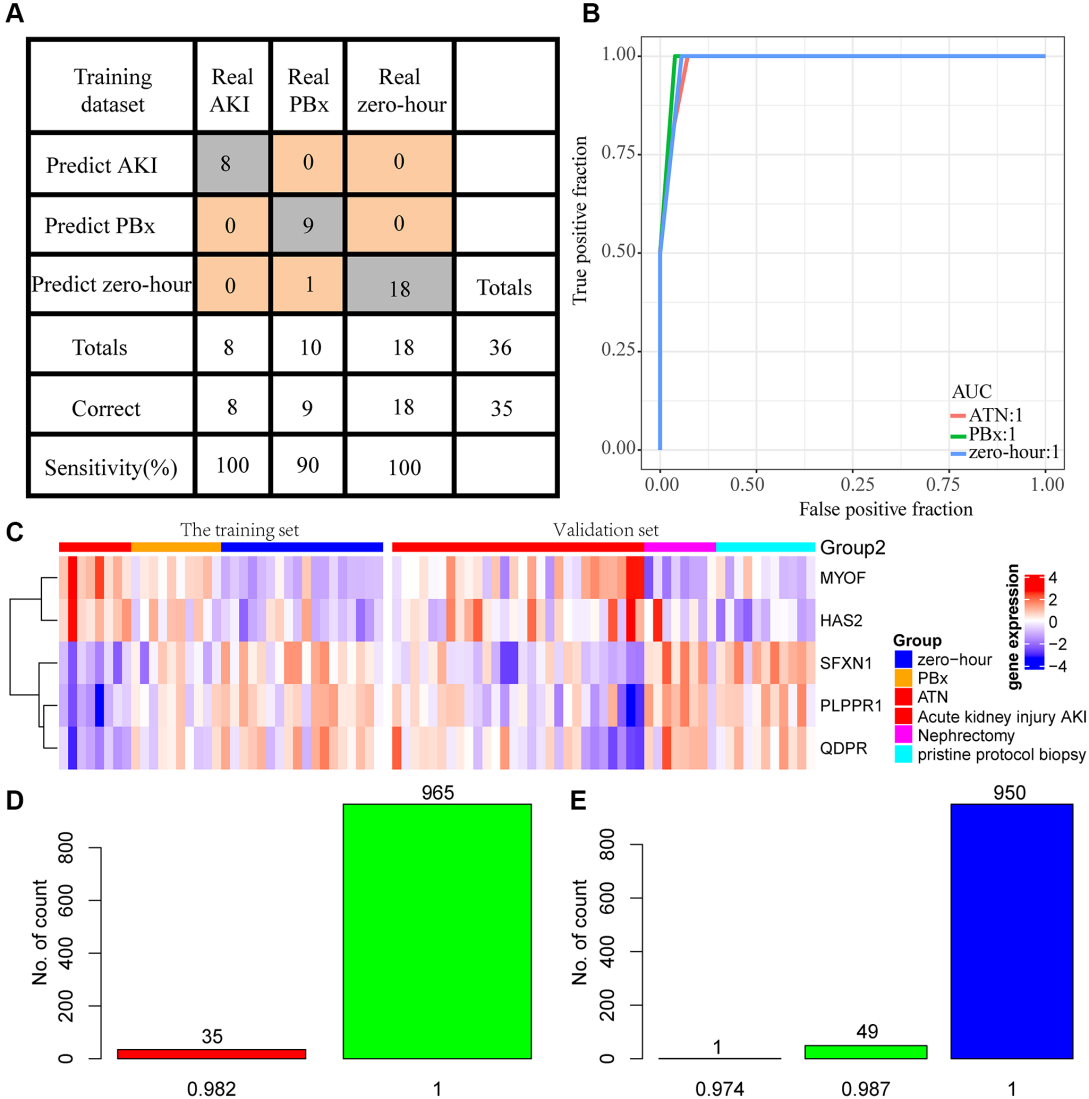
**Construction of diagnostic model.** (**A**) Prediction of AKI, PBx, and zero-hour in a diagnostic model constructed from 5 genes. (**B**) ROC curves for the prediction of AKI, PBx, and zero-hour in a diagnostic model constructed by 5 genes. (**C**) Expression patterns of the five genes in the training set and external test sets. (**D**) A thousand randomized validations of the distribution of predictive accuracy for nephrectomy samples and AKI samples in the validation set. (**E**) Thousands of random verifications in the verification set for the prediction accuracy distribution of stable kidney transplant samples and AKI samples.

Studies have increasingly shown that combining multiple genes is more predictive than the use of a single gene, therefore the five genes with the highest AUC were selected. The gene expression profiling of 36 samples was used as the training set (AKI = 8, PBx = 10, zero-hour = 18) to construct the classification model with support vector machine. The model was tested by ten-fold cross validation method. The classification accuracy of AKI was 100%, that of PBx was 90%, and that of zero-hour was 94.7% (Figure 5A). Among the 36 samples, 35 were correctly classified, and the AUC of the 3 samples was 1 (Figure 5B), indicating that these 5 genes could accurately predict AKI, and distinguish AKI from other chronic kidney diseases. Next, the model was applied to the external verification data set GSE30718 and GSE30718. The expression patterns of the five genes in the verification set were found to be highly consistent with the training set. MYOF and HAS2 were high-expressed in AKI, and SFXN1, PLPPR1, and QDPR were low-expressed in AKI (Figure 5C). 8 nephrectomy samples and randomly selected 8 AKI samples repeated for 1000 times were used as a verification set and substituted into the model for further verifying the prediction accuracy of the model. The prediction accuracy rate was 100% and 98% when repeated for 965 times and 35 times, respectively (Figure 5D). In addition, we selected 11 samples of stable kidney transplantation and randomly selected 1000 AKI samples as a verification set to verify the model performance, the prediction accuracy rate of which was 100%, 98.7%, and 97.4% when repeated for 950 times (95%), 49 times (4.9 %), and 1 time (0.1%), respectively (Figure 5E). Moreover, GSE37838 dataset with 70 samples [12] containing 12 AKI samples and 58 immediate graft function (IGF) samples were obtained for validation. The expression profiles of HAS2, MYOF, PLPPR1, QDPR, and SFXN1 genes were extracted from GSE37838, and we found that HAS2 and MYOF expression was upregulated in AKI, and that PLPPR1, QDPR, and SFXN1 genes were significantly downregulated in AKI, which was consistent with our results (Supplementary Figure 1B–1C). These results indicated that the five-gene model had a high prediction performance.

### Potential regulatory pathways of the five-gene diagnosis model

**Figure 6:**
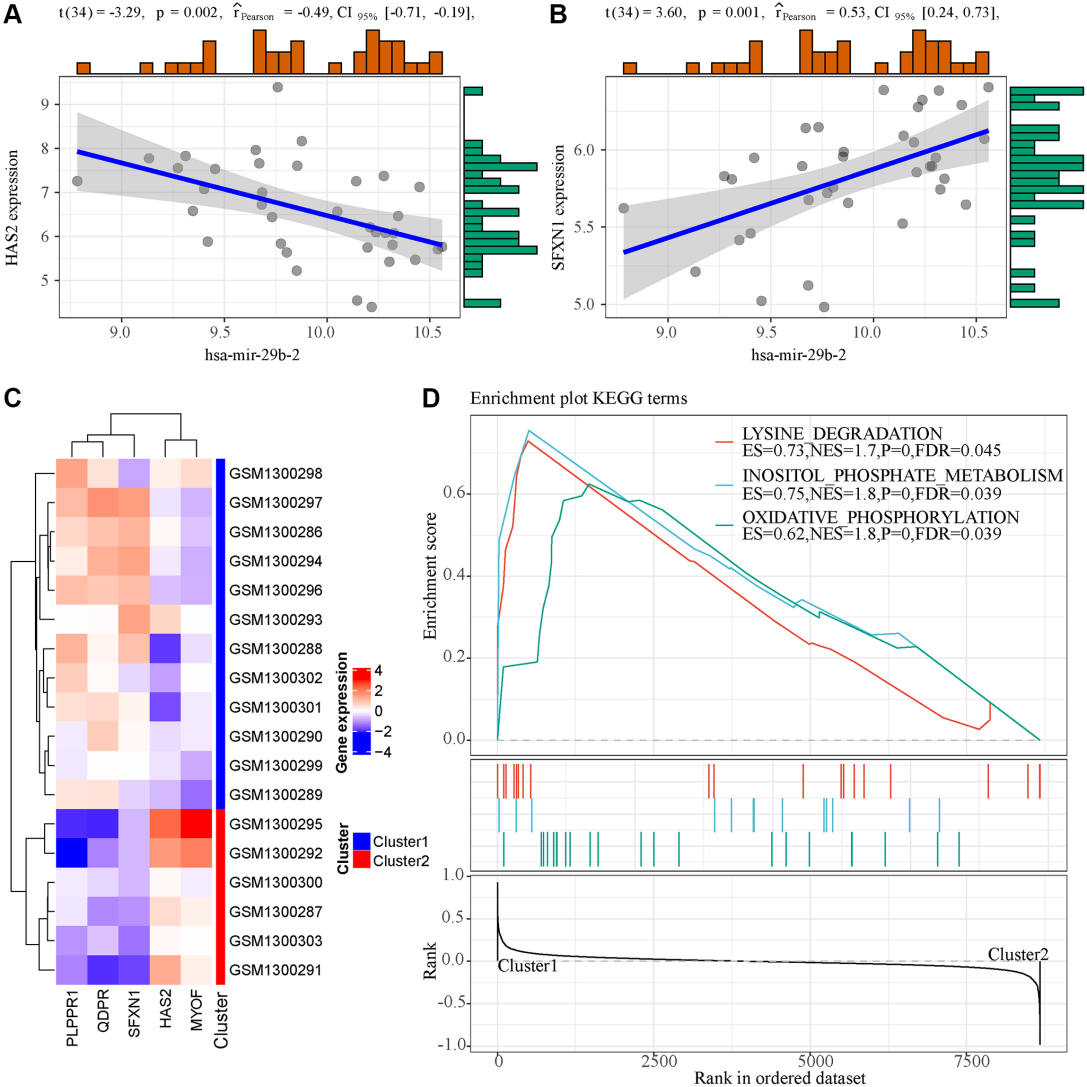
**Functional analysis of 5 genes in the model.** (**A**) The expression of hsa-mir-29b-2 was correlated with HAS2. (**B**) The expression of hsa-mir-29b-2 was correlated with SFXN1. (**C**) Unsupervised clustering of five genes in the model. (**D**) GSEA enrichment analysis results of two samples with different expression patterns.

To observe the potential regulatory patterns of these genes, we first separately predicted the miRNAs targeting these genes. In combination with the phenotype-associated miRNA co-expression module, it was observed that hsa-mir-29b-2 targeted HAS2 and SFXN1 in the four core miRNAs of the miRNA co-expression module, and interestingly hsa-mir-29b-2 was significantly negatively associated with HAS2 (Figure 6A) but positively correlated with SFXN1 (Figure 6B). This indicated that mir-29b-2 may be involved in the occurrence and development of AKI. Furthermore, based on the expression of these five genes, unsupervised clustering was conducted on zero-hour samples, and the results showed that these five genes could be divided into two groups (Cluster1 and Cluster2) (Figure 6C), where HAS2 and MYOF were high-expressed in Cluster1 and PLPPR1, QDPR, and SFXN1 were low-expressed in Cluster1. After analyzing the differences in the KEGG pathways between the two samples with GSEA (Figure 6D), three pathways, namely, LYSINE DEGRADATION, INOSITOL PHOSPHATE METABOLISM and oxidation, were observed to be significantly enriched in Cluster1, suggesting that there may be phosphorylation abnormalities in AKI-like samples, which were indirectly or directly involved in the regulation of AKI.

## DISCUSSION

AKI is a common complication of kidney transplantation and is associated with a shorter graft survival [13, 14]. AKI, which is usually diagnosed based on an increase in serum creatinine than the preoperative level, commonly occurs several days after initial injury. Identifying high-risk individuals with the risk of developing AKI quickly after surgery can improve the prognosis of patients, and is considered an important step in the preventing postoperative AKI [15]. In this study, the expression patterns of genes and miRNAs were compared between the newly transplanted samples and those 24 hours after transplantation with weighted co-expression analysis. 17 genes, including HAS2, MYOF, PLPPR1, QDPR and SFXN1, were determined as early diagnostic markers for AKI. Their AUC ranged from 0.82-0.96, showing a high predictive performance for AKI samples. Meanwhile, functional analysis also demonstrated that these genes were mainly related to transmembrane transport, amino acid metabolism, renal absorption and positive regulation of urine volume. Studies reported that transmembrane transport and amino acid metabolism play important roles in patients with acute renal failure [16, 17], indicating that these genes may have critical regulatory functions in the occurrence and development of AKI.

Biomarkers of kidney injury have the potential of non-invasive assessment of graft injury, and a variety of proteins, for example, neutrophil gelatinase-associated lipocalin (NGAL), IL-18, which are released into the urine during renal tubule cell injury, have been identified for quantitative ischemia/reperfusion injury to the kidney, moreover, NGAL and IL-18 are detectable by non-invasive methods [18, 19]. The availability of biomarkers such as NGAL and il-18 allows noninvasive assessments of early graft damage to facilitate clinical decision-making and potentially protect long-term graft function [19, 20]. In this study, the performance of NGAL and il-18 in predicting AKI was analyzed, and the AUC reached 0.78 and 0.8, respectively (Supplementary Figure 2A). In comparison, the AUC of the 17 genes showed a high predictive performance, suggesting that NGAL and il-18 may not be the most indicative markers in tissue samples. As the prediction accuracy of multi-gene model is often higher than the use of a single gene, we selected five genes (HAS2, MYOF, PLPPR1, QDPR and SFXN1) with the highest AUC. The method of support vector machine was used to establish a diagnostic model of AKI, surprisingly, these five genes showed a significantly high AUC in the training set and verification set, reaching an AKI prediction accuracy of 100%. To verify whether the five-gene signature could predict patients’ risk of developing AKI earlier before kidney transplantation, we selected samples collected at 0 hour of transplantation to predict the risk of AKI 24 hours later. It was found that these 5 genes were 100% accurate in prediction (Supplementary Figure 2B), showing that these five genes may serve as biomarkers for the development of AKI after kidney transplantation as well as for the development of transplantation drugs to guide clinical trials.

In addition, several of these five genes have been reported to be associated with kidney diseases, for instance, the renal fibrosis and hyaluronic acid (HA) is associated with increased cortical synthesis, human hyaluronic acid synthase 2 (HAS2) transcription induction, and subsequent HAS2-driven HA synthesis may adjust the phenotype of renal proximal renal tubular epithelial cells (PTC) and result in renal fibrosis [21]. The endothelial loss of hyaluronic acid leads to the destruction of glomerular endothelial stability, which will affect glomerular function and structural integrity [22]. MYOF is a prognostic marker in clear cell renal cell carcinoma [23]. Myoferlin hyperexpression has been determined as an independent risk factor in developing a subsequent primary malignant tumor in patients with ccRCC [24]. QDPR may be an important factor in mediating diabetic nephropathy through regulating TGF- TGF 1/ Smad3 signaling and NADPH oxidase [25]. Overexpression of QDPR in HEK293T cells in human kidney significantly protects against oxidative stress [26]. Although PLPPR1 and SFXN1 genes have not been reported to be associated with kidney disease, the current study found that PLPPR1 and SFXN1 were significantly positively correlated with QDPR and negatively correlated with HAS2 and MYOF. GSEA analysis showed that a high expression of PLPPR1 and SFXN1 may be associated with the activation of LYSINE DEGRADATION, INOSITOL PHOSPHATE METABOLISM and OXIDATIVE DEGRADATION pathways. These results demonstrated that the diagnostic model developed based on these genes had a clinical application potential and could facilitate the diagnosis of clinical patients and drug development.

Although bioinformatics analyses were used to identify potential candidate genes for AKI in large samples, some limitations of this study should be noted. Firstly, the samples lacked some clinical follow-up information, excluding the possibility of differentiating diagnostic biomarkers by taking into account factors such as the presence of other health conditions of patients. Secondly, the study lacked follow-up data, systematic assessment the influence of these genes on the prognosis of renal transplant patients was not possible. Moreover, the results obtained only by bioinformatics analysis were not sufficiently convincing, which requires further experimental verification. Therefore, it is necessary to conduct further verification and experimental research with larger sample size and experiments.

## CONCLUSIONS

In summary, in this study, we identified 17 AKI-related genes and developed a 5-gene signature for the diagnosis and prediction of AKI. It has a high AUC in both the training set and the validation set, and better predictive performance compared with using NGAL and IL-18 in AKI detection.

## MATERIALS AND METHODS

### Data collection

**Figure 7:**
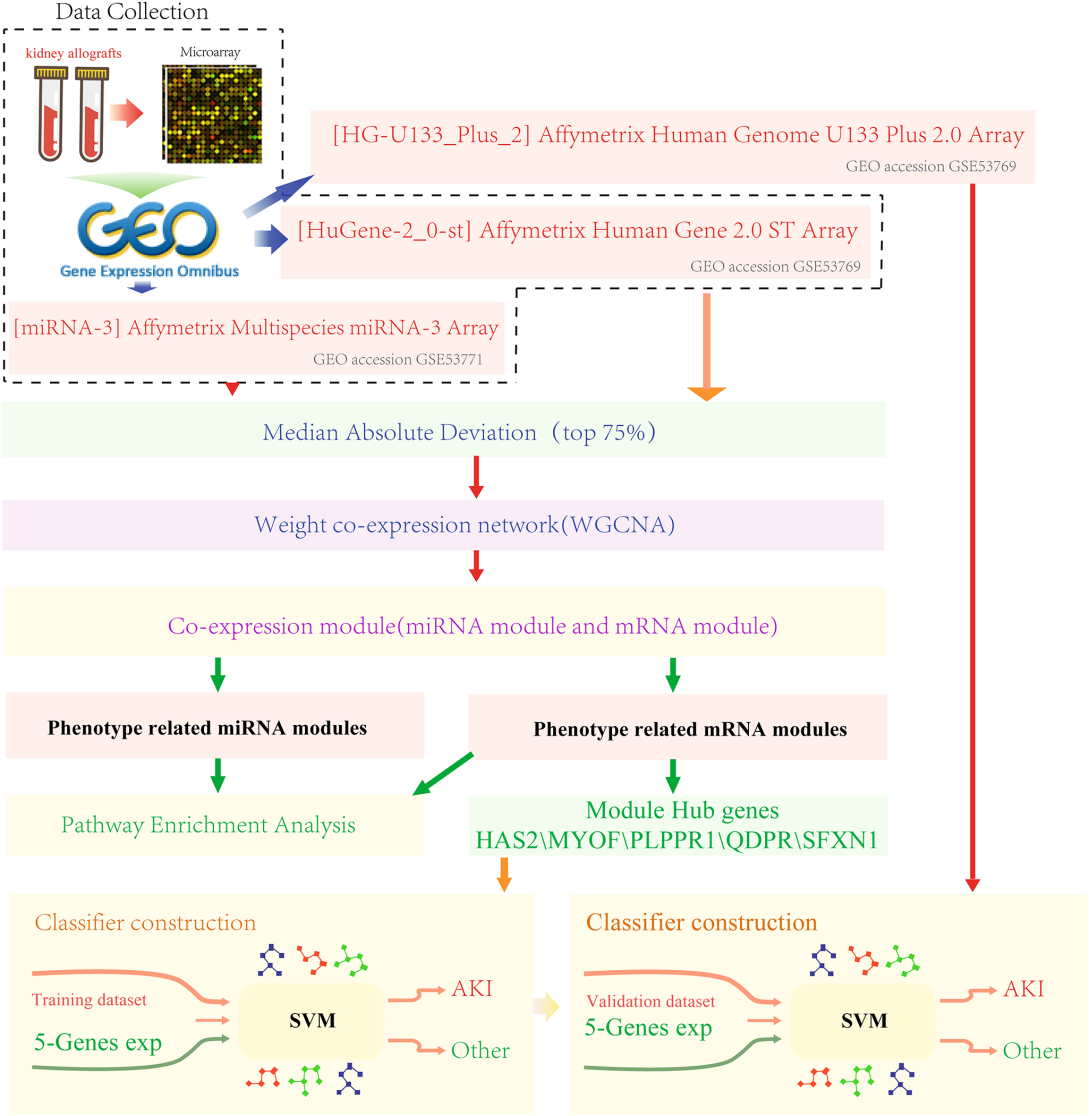
Work flow chart.

In this study, we screened two sets of gene expression data and one set of miRNA expression data from the Gene Expression Omnibus (GEO) database (http://www.ncbi.nlm.nih.gov/geo/). miRNA expression data was from the GPL16384 platform ([miRNA-3] Affymetrix Multispecies miRNA-3 Array, dataset ID GSE53771) [27, 28]. Gene expression profiling data were collected from two platforms. The platform for the dataset GSE53769 [27, 28] was the GPL16686 platform ([HuGene-2_0-st] Affymetrix Human Gene 2.0 ST Array [transcript (gene) version]), and that for dataset GSE30718 was the GPL570 platform ([HG-U133_Plus_2] Affymetrix Human Genome U133 Plus 2.0 Array). Sample distribution for each dataset was shown in Table 1. The GSE53771 and GSE53769 datasets were derived from preoperative and postoperative samples of 18 patients treated with immunosuppressive therapy, and postoperative samples were follow-up samples with kidney biopsies performed within 12 days of transplantation. Specifically, eight patients with acute tubular necrosis without rejection were defined as AKI, while ten protocol samples with biopsies but without pathology were in control group (primary graft function). Patients with AKI were identified according to the Banff 2012 criteria [29]. AKI kidney biopsies were indicated by more than one dialysis session during the first week after transplantation, with serum sarcosine higher than 4 mg/dL the first week after surgery. The GSE30718 dataset was derived from 39 samples of post-transplant of kidney, all the patients were treated with immunosuppressive therapy, and biopsies were obtained one year after transplantation (other clinical therapies were administered as symptomatic treatment, detailed clinical information was shown in Supplementary Table 1). The work flow chart was shown in Figure 7.

**Table 1:** Sample distribution of each data set.

**GEO Accession**	**No. of AKI**	**No. of PBx**	**No. of control**	**No. of Nephrectomy**
**GSE53771**	8	10	18	10
**GSE53769**	8	10	18	9
**GSE30718**	28	0	11	8

### Data processing

For gene expression data, the probes of the standardized chip data downloaded were annotate by the R package hgu133plus2.db. The probes that were matched to multiple genes were removed, while the median of those matched to a gene was regarded as the expression of the modified genes. Finally, expression profiles of 8675 genes and 36 samples were extracted from the GSE53769 data set, and 47 samples of 20,549 genes were obtained from the GSE30718 data set. For miRNA expression data, we downloaded the standardized microarray data and obtained miRNA expression data of 1239 miRNAs and 36 samples from other human species.

### Co-expression network construction

Firstly, mRNA/miRNA data profile of top75% MADs was validated for sample and miRNA/ mRNA quality. Then, the WGCNA [30] package in R was used to construct a scale-free co-expression network for the mRNA/miRNAs. First, the Pearson’s correlation matrices and average linkage method were performed for all pair-wise mRNA/miRNAs. Then, a weighted adjacency matrix was constructed using a power function A_*mn*_ = |C_*mn*_|^*β*^ (C_*mn*_ = Pearson’s correlation between mRNA/miRNA m and mRNA/miRNA n; A_*mn*_ = adjacency between mRNA/miRNA m and mRNA/miRNA n). β was a soft-thresholding parameter emphasizing strong correlations between mRNA/miRNAs and penalizing weak correlations. After choosing the power of β, the adjacency was transformed into a topological overlap matrix (TOM) to measure the network connectivity of an mRNA/miRNA, which was defined as the sum of its adjacency with all other mRNA/miRNAs, and the corresponding dissimilarity (1-TOM) was calculated. To classify mRNA/miRNAs with similar expression profiles into mRNA/miRNA modules, average linkage hierarchical clustering was conducted for the mRNA/miRNAs dendrogram, according to the TOM-based dissimilarity measured with a minimum size (mRNA/miRNA group) of 30. To further analyze the module, we calculated the dissimilarity of module mRNA/miRNAs to determine a cut line for module dendrogram and merged some module.

### Gene set enrichment analysis

Gene Set Enrichment Analysis (GSEA) [31] was performed by the JAVA program (http://software.broadinstitute.org/gsea/downloads.jsp) using the MSigDB [32] on C2 Canonical pathways gene set collection containing 1320 gene sets. Gene sets with an FDR less than 0.01 after performing 1000 permutations were considered to be significantly enriched.

### Functional enrichment analyses

Gene Ontology (GO) and Kyoto Encyclopedia of Genes and Genomes (KEGG) pathway enrichment analysis was conducted in the R package clusterProfiler [33] to identify over-represented GO terms in three categories (biological processes, molecular function and cellular component), and KEGG pathway. Here, a *p* < 0.05 was considered to denote statistical significance.

### Correlation analysis between module and phenotype, and identification of hub gene

To examine the correlation between co-expression modules and phenotypes, we defined a 0-1 matrix of phenotypes. Specifically, for any phenotype such as AKI, a correct detection of AKI from the samples was marked as 1, otherwise it is marked as 0. In this way, the numeric matrix of each phenotype was established, and the correlation between the feature vector of each module and the numeric matrix of the phenotype was calculated using the Spielman rank correlation coefficient, with *p* < 0.05 indicating a significant phenotype in the module. Two methods were used to determine hub genes. Firstly, the correlation between the expression of each gene and the module feature vector was calculated with correlation coefficient was greater than 0.8.

### Prediction of miRNA target genes

Experimentally verified miRNA target gene databases miRecords [34], miRTarBase [35] and starBase [36] were applied to identify reliable miRNA target genes.

### Construction of diagnostic prediction model and evaluation of model performance

Feature genes were used to construct a diagnostic prediction model based on SVM [37] classification to predict AKI and non-AKI samples. Support vector machine (SVM), which is a supervised learning model in machine learning algorithm, analyzes data and identifies patterns. A support vector mechanism creates a hyperplane in a high or infinite dimensional space for classification and regression. Given a set of training samples, each mark belongs to two classes. A SVM training algorithm establishes a model and assigns new instances to one class or another, making it a binary linear classification. We built the model in the training data set and used the ten-fold cross validation method to verify the classification ability of the model. The established model was then used to predict the samples in the validation data set. The predictive power of the model was evaluated using the area under the ROC curve (AUC), and the sensitivity and specificity of the model to AKI were analyzed.

### The model prediction ability in an external data set

GSE30718 served as an independent external validation dataset, and we downloaded the normalized data, extracted the expression levels of the trait genes and proxied the model against the samples to verify the accuracy of the model prediction.

### Statistical analysis

The R package pROC was used for AUC analysis, and the R package ComplexHeatmap was used for heat map drawing. All analyses, USES default parameters, and data visualization were performed using ggplot2 in version 3.4.3 of R software, if not specially instructed.

## Supplementary Materials

Supplementary Figures

Supplementary Table 1

## References

[1] Bonventre JV, Yang L. Cellular pathophysiology of ischemic acute kidney injury. J Clin Invest. 2011; 121:4210–21. 10.1172/JCI45161 22045571 PMC3204829

[2] Srisawat N, Kellum JA. Acute kidney injury: definition, epidemiology, and outcome. Curr Opin Crit Care. 2011; 17:548–55. 10.1097/MCC.0b013e32834cd349 22027404

[3] Bellomo R, Kellum JA, Ronco C. Acute kidney injury. Lancet. 2012; 380:756–66. 10.1016/S0140-6736(11)61454-2 22617274

[4] Chertow GM, Burdick E, Honour M, Bonventre JV, Bates DW. Acute kidney injury, mortality, length of stay, and costs in hospitalized patients. J Am Soc Nephrol. 2005; 16:3365–70. 10.1681/ASN.2004090740 16177006

[5] Haase M, Shaw A. Acute kidney injury and cardiopulmonary bypass: special situation or same old problem? Contrib Nephrol. 2010; 165:33–8. 10.1159/000313742 20427953

[6] Lafrance JP, Miller DR. Acute kidney injury associates with increased long-term mortality. J Am Soc Nephrol. 2010; 21:345–52. 10.1681/ASN.2009060636 20019168 PMC2834549

[7] Bagshaw SM, George C, Bellomo R, and ANZICS Database Management Committe. A comparison of the RIFLE and AKIN criteria for acute kidney injury in critically ill patients. Nephrol Dial Transplant. 2008; 23:1569–74. 10.1093/ndt/gfn009 18281319

[8] Bellomo R, Kellum JA, Ronco C. Defining acute renal failure: physiological principles. Intensive Care Med. 2004; 30:33–7. 10.1007/s00134-003-2078-3 14618231

[9] Parikh CR, Thiessen-Philbrook H, Garg AX, Kadiyala D, Shlipak MG, Koyner JL, Edelstein CL, Devarajan P, Patel UD, Zappitelli M, Krawczeski CD, Passik CS, Coca SG, and TRIBE-AKI Consortium. Performance of kidney injury molecule-1 and liver fatty acid-binding protein and combined biomarkers of AKI after cardiac surgery. Clin J Am Soc Nephrol. 2013; 8:1079–88. 10.2215/CJN.10971012 23599408 PMC3700701

[10] Meersch M, Schmidt C, Van Aken H, Martens S, Rossaint J, Singbartl K, Görlich D, Kellum JA, Zarbock A. Urinary TIMP-2 and IGFBP7 as early biomarkers of acute kidney injury and renal recovery following cardiac surgery. PLoS One. 2014; 9:e93460. 10.1371/journal.pone.0093460 24675717 PMC3968141

[11] Zou YF, Wen D, Zhao Q, Shen PY, Shi H, Zhao Q, Chen YX, Zhang W. Urinary MicroRNA-30c-5p and MicroRNA-192-5p as potential biomarkers of ischemia-reperfusion-induced kidney injury. Exp Biol Med (Maywood). 2017; 242:657–67. 10.1177/1535370216685005 28056546 PMC5685255

[12] Kreepala C, Famulski KS, Chang J, Halloran PF. Comparing molecular assessment of implantation biopsies with histologic and demographic risk assessment. Am J Transplant. 2013; 13:415–26. 10.1111/ajt.12043 23282320

[13] Hariharan S, McBride MA, Cherikh WS, Tolleris CB, Bresnahan BA, Johnson CP. Post-transplant renal function in the first year predicts long-term kidney transplant survival. Kidney Int. 2002; 62:311–8. 10.1046/j.1523-1755.2002.00424.x 12081593

[14] Yarlagadda SG, Coca SG, Formica RN Jr, Poggio ED, Parikh CR. Association between delayed graft function and allograft and patient survival: a systematic review and meta-analysis. Nephrol Dial Transplant. 2009; 24:1039–47. 10.1093/ndt/gfn667 19103734

[15] Thiele RH, Isbell JM, Rosner MH. AKI associated with cardiac surgery. Clin J Am Soc Nephrol. 2015; 10:500–14. 10.2215/CJN.07830814 25376763 PMC4348689

[16] Abel RM, Shih VE, Abbott WM, Beck CH Jr, Fischer JE. Amino acid metabolism in acute renal failure: influence of intravenous essential L-amino acid hyperalimentation therapy. Ann Surg. 1974; 180:350–5. 10.1097/00000658-197409000-00016 4850497 PMC1343671

[17] Neri M, Villa G, Garzotto F, Bagshaw S, Bellomo R, Cerda J, Ferrari F, Guggia S, Joannidis M, Kellum J, Kim JC, Mehta RL, Ricci Z, et al, and Nomenclature Standardization Initiative (NSI) alliance. Nomenclature for renal replacement therapy in acute kidney injury: basic principles. Crit Care. 2016; 20:318. 10.1186/s13054-016-1489-9 27719682 PMC5056503

[18] Gracie JA, Robertson SE, McInnes IB. Interleukin-18. J Leukoc Biol. 2003; 73:213–24. 10.1189/jlb.0602313 12554798

[19] Paragas N, Qiu A, Zhang Q, Samstein B, Deng SX, Schmidt-Ott KM, Viltard M, Yu W, Forster CS, Gong G, Liu Y, Kulkarni R, Mori K, et al. The Ngal reporter mouse detects the response of the kidney to injury in real time. Nat Med. 2011; 17:216–22. 10.1038/nm.2290 21240264 PMC3059503

[20] Hall IE, Koyner JL, Doshi MD, Marcus RJ, Parikh CR. Urine cystatin C as a biomarker of proximal tubular function immediately after kidney transplantation. Am J Nephrol. 2011; 33:407–13. 10.1159/000326753 21494031 PMC3100377

[21] Michael DR, Phillips AO, Krupa A, Martin J, Redman JE, Altaher A, Neville RD, Webber J, Kim MY, Bowen T. The human hyaluronan synthase 2 (HAS2) gene and its natural antisense RNA exhibit coordinated expression in the renal proximal tubular epithelial cell. J Biol Chem. 2011; 286:19523–32. 10.1074/jbc.M111.233916 21357421 PMC3103331

[22] van den Berg BM, Wang G, Boels MGS, Avramut MC, Jansen E, Sol WMP, Lebrin F, van Zonneveld AJ, de Koning EJP, Vink H, Gröne HJ, Carmeliet P, van der Vlag J, Rabelink TJ. Glomerular Function and Structural Integrity Depend on Hyaluronan Synthesis by Glomerular Endothelium. J Am Soc Nephrol. 2019; 30:1886–97. 10.1681/ASN.2019020192 31308073 PMC6779367

[23] Jung M, Lee C, Park JH, Moon KC. Prognostic significance of immunohistochemical staining for myoferlin in clear cell renal cell carcinoma and its association with epidermal growth factor receptor expression. Urol Oncol. 2019; 37:812.e9–e16. 10.1016/j.urolonc.2019.07.002 31421995

[24] Koh HM, An HJ, Ko GH, Lee JH, Lee JS, Kim DC, Seo DH, Song DH. Identification of Myoferlin Expression for Prediction of Subsequent Primary Malignancy in Patients With Clear Cell Renal Cell Carcinoma. In Vivo. 2019; 33:1103–8. 10.21873/invivo.11579 31280198 PMC6689344

[25] Gu Y, Gong Y, Zhang H, Dong X, Zhao T, Burczynski FJ, Wang G, Sun S, Zhu B, Han W, Wang H, Li P. Regulation of transforming growth factor beta 1 gene expression by dihydropteridine reductase in kidney 293T cells. Biochem Cell Biol. 2013; 91:187–93. 10.1139/bcb-2012-0087 23668792

[26] Si Q, Sun S, Gu Y. A278C mutation of dihydropteridine reductase decreases autophagy via mTOR signaling. Acta Biochim Biophys Sin (Shanghai). 2017; 49:706–12. 10.1093/abbs/gmx061 28633336

[27] Korbély R, Wilflingseder J, Perco P, Kainz A, Langer RM, Mayer B, Oberbauer R. Molecular biomarker candidates of acute kidney injury in zero-hour renal transplant needle biopsies. Transpl Int. 2011; 24:143–9. 10.1111/j.1432-2277.2010.01162.x 20819195

[28] Wilflingseder J, Sunzenauer J, Toronyi E, Heinzel A, Kainz A, Mayer B, Perco P, Telkes G, Langer RM, Oberbauer R. Molecular pathogenesis of post-transplant acute kidney injury: assessment of whole-genome mRNA and miRNA profiles. PLoS One. 2014; 9:e104164. 10.1371/journal.pone.0104164 25093671 PMC4122455

[29] Solez K, Colvin RB, Racusen LC, Haas M, Sis B, Mengel M, Halloran PF, Baldwin W, Banfi G, Collins AB, Cosio F, David DS, Drachenberg C, et al. Banff 07 classification of renal allograft pathology: updates and future directions. Am J Transplant. 2008; 8:753–60. 10.1111/j.1600-6143.2008.02159.x 18294345

[30] Langfelder P, Horvath S. WGCNA: an R package for weighted correlation network analysis. BMC Bioinformatics. 2008; 9:559. 10.1186/1471-2105-9-559 19114008 PMC2631488

[31] Subramanian A, Kuehn H, Gould J, Tamayo P, Mesirov JP. GSEA-P: a desktop application for Gene Set Enrichment Analysis. Bioinformatics. 2007; 23:3251–3. 10.1093/bioinformatics/btm369 17644558

[32] Liberzon A, Subramanian A, Pinchback R, Thorvaldsdóttir H, Tamayo P, Mesirov JP. Molecular signatures database (MSigDB) 3.0. Bioinformatics. 2011; 27:1739–40. 10.1093/bioinformatics/btr260 21546393 PMC3106198

[33] Yu G, Wang LG, Han Y, He QY. clusterProfiler: an R package for comparing biological themes among gene clusters. OMICS. 2012; 16:284–7. 10.1089/omi.2011.0118 22455463 PMC3339379

[34] Xiao F, Zuo Z, Cai G, Kang S, Gao X, Li T. miRecords: an integrated resource for microRNA-target interactions. Nucleic Acids Res. 2009; 37:D105–10. 10.1093/nar/gkn851 18996891 PMC2686554

[35] Huang HY, Lin YC, Li J, Huang KY, Shrestha S, Hong HC, Tang Y, Chen YG, Jin CN, Yu Y, Xu JT, Li YM, Cai XX, et al. miRTarBase 2020: updates to the experimentally validated microRNA-target interaction database. Nucleic Acids Res. 2020; 48:D148–54. 10.1093/nar/gkz896 31647101 PMC7145596

[36] Vergoulis T, Vlachos IS, Alexiou P, Georgakilas G, Maragkakis M, Reczko M, Gerangelos S, Koziris N, Dalamagas T, Hatzigeorgiou AG. TarBase 6.0: capturing the exponential growth of miRNA targets with experimental support. Nucleic Acids Res. 2012; 40:D222–9. 10.1093/nar/gkr1161 22135297 PMC3245116

[37] Sanz H, Valim C, Vegas E, Oller JM, Reverter F. SVM-RFE: selection and visualization of the most relevant features through non-linear kernels. BMC Bioinformatics. 2018; 19:432. 10.1186/s12859-018-2451-4 30453885 PMC6245920

