# Supplementary material for: Five-gene signature predicts acute kidney injury in early kidney transplant patients
Source: Aging (Albany NY). 2022 Mar 23;14(6):2628–44. doi: 10.18632/aging.203962 (PMC9004575; doi:10.18632/aging.203962)
Supplement: Supplementary Figures [file aging-14-203962-s001.pdf]

SUPPLEMENTARY FIGURES

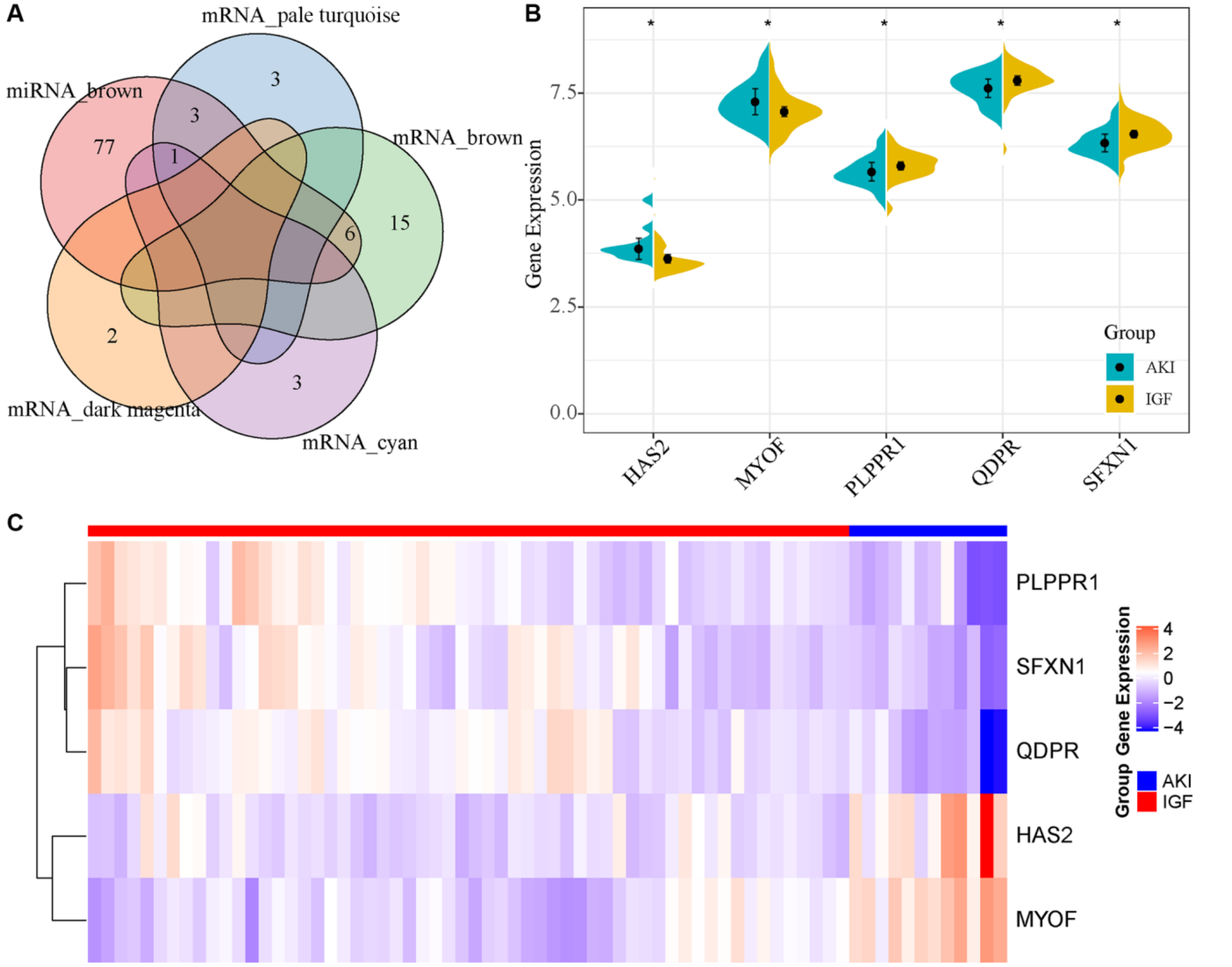

Supplementary Figure 1. Venn diagrams and dataset validation. (**A**) The phenotypic related modules were enriched into the intersection of the KEGG Pathway in the Venn diagram. (**B**) The expression distribution of five genes in GSE30718 dataset. (**C**) Heat map of expression of five genes in GSE30718 dataset.

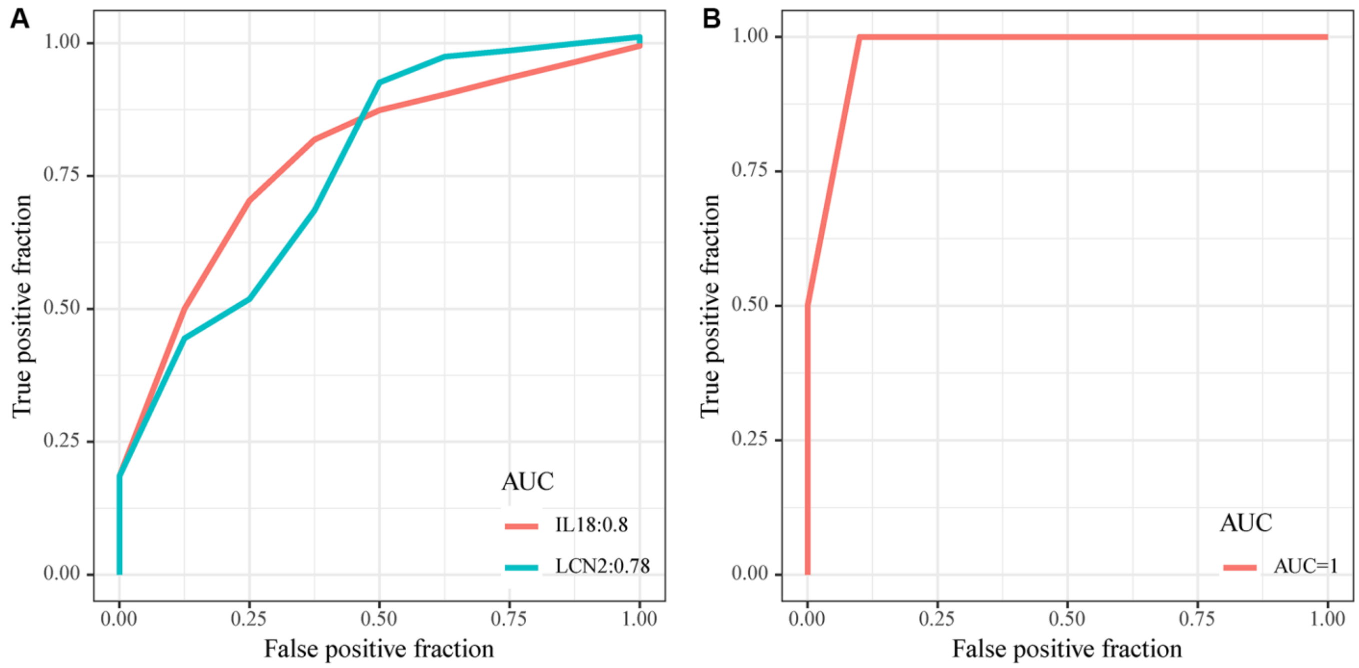

Supplementary Figure 2. AUC analysis of NGAL and IL-18. (**A**) ROC curve predicted by NGAL and IL-18 gene expression against AKI samples. (**B**) ROC curve of 5 gene signatures in the 0-hour sample predicted the 24-hour AKI.
